# Multispectral and Colorimetric Approaches for Non-Destructive Maturity Assessment of Specialty Arabica Coffee

**DOI:** 10.3390/foods14213644

**Published:** 2025-10-25

**Authors:** Seily Cuchca Ramos, Jaris Veneros, Carlos Bolaños-Carriel, Grobert A. Guadalupe, Marilu Mestanza, Heyton Garcia, Segundo G. Chavez, Ligia Garcia

**Affiliations:** 1 Maestría en Cambio Climático, Agricultura y Desarrollo Rural Sostenible (MACCARD), Escuela de Posgrado (EPG), Universidad Nacional Toribio Rodríguez de Mendoza (UNTRM), Chachapoyas 01001, Peru; seily.cuchca.epg@untrm.edu.pe; 2 Instituto de Investigación para el Desarrollo Sustentable de Ceja de Selva (Indes-Ces), Universidad Nacional Toribio Rodríguez de Mendoza de Amazonas, 342 Higos Urco, Chachapoyas 01001, Peru; jaris.veneros@untrm.edu.pe (J.V.); grobert.guadalupe@untrm.edu.pe (G.A.G.); heyton.garcia.epg@untrm.edu.pe (H.G.); segundo.quintana@untrm.edu.pe (S.G.C.); 3 Laboratorio de Microbiología y Fitopatología, Facultad de Ciencias Agrícolas, Universidad Central del Ecuador, Quito 170521, Ecuador; cabolanosc@uce.edu.ec; 4 Facultad de Ingeniería Zootecnista, Biotecnología, Agronegocios y Ciencia de Datos, Universidad Nacional Toribio Rodríguez de Mendoza de Amazonas, 342 Higos Urco, Chachapoyas 01001, Peru; marilu.mestanza@untrm.edu.pe

**Keywords:** *Coffea arabica*, colorimetry, remote sensing, principal component analysis (PCA), multiple linear regression, maturity

## Abstract

This study evaluated the integration of non-invasive remote sensing and colorimetry to classify the maturity stages of Coffea arabica fruits across four varieties: Caturra Amarillo, Excelencia, Milenio, and Típica. Multispectral signatures were captured using a Parrot Sequoia camera at wavelengths of 550 nm, 660 nm, 735 nm, and 790 nm, while colorimetric parameters L*, a*, and b* were measured with a high-precision colorimeter. We conducted multivariate analyses, including Principal Component Analysis (PCA) and multiple linear regression (MLR), to identify color patterns and develop predictors for fruit maturity. Spectral curve analysis revealed consistent changes related to ripening: a decrease in reflectance in the green band (550 nm), a progressive increase in the red band (660 nm), and relative stability in the RedEdge and near-infrared regions (735–790 nm). Colorimetric analysis confirmed systematic trends, indicating that the a* component (green to red) was the most reliable indicator of ripeness. Additionally, L* (lightness) decreased with maturity, and the b* component (yellowness to blue) showed varying importance depending on the variety. PCA accounted for over 98% of the variability across all varieties, demonstrating that these three parameters effectively characterize maturity. MLR models exhibited strong predictive performance, with adjusted R^2^ values ranging between 0.789 and 0.877. Excelencia achieved the highest predictive accuracy, while Milenio demonstrated the lowest, highlighting varietal differences in pigmentation dynamics. These findings show that combining multispectral imaging, colorimetry, and statistical modeling offers a non-destructive, accessible, and cost-effective method for objectively classifying coffee maturity. Integrating this approach into computer vision or remote sensing systems could enhance harvest planning, reduce variability in specialty coffee lots, and improve competitiveness by ensuring greater consistency in cup quality.

## 1. Introduction

Coffee is one of the most valuable agricultural products and plays a crucial role in generating foreign exchange earnings for many countries [1]. Its commercial value is directly tied to the quality of the beverage produced [2]. Green coffee beans, the primary item in international trade [3], are assessed based on various quality criteria [4]. These beans can have defects such as black beans, sour beans, fungal damage, foreign matter, dry cherries, insect damage, split or bitten beans, cut beans, immature beans, overripe beans, shells, floaters, parchment, and husks [5]. The presence of immature beans increases the astringency of the beverage and decreases its overall sensory quality [6], leading to economic losses for producers. For coffee to be classified and sold as specialty coffee, it must meet several specific criteria, including the absence of Quaker beans, which are immature beans that do not develop during the roasting process [5]. Additionally, the ripeness of the fruit at harvest time influences the physicochemical properties of the beans, thereby affecting their quality [7]. Improving coffee quality is essential for increasing its market value [8].

Artificial vision systems for agriculture have emerged as a valuable tool for selecting and classifying agricultural products based on their colorimetric characteristics [9,10]. Research on artificial vision for coffee aims to gather information that can assist in non-invasive harvesting [11]. Defective coffee beans typically result from issues during harvesting and pre-processing [12]. Identifying unripe beans before processing could improve the quality of specialty coffee and minimize contamination risks. Systems that employ optical techniques can measure parameters without damaging the fruit and can be integrated into automated equipment [13]. These systems provide highly accurate results that can be compared to human sensory evaluation methods [14]. Unripe beans are often found in large quantities, particularly in large-scale harvesting operations aimed at bulk sales. While electronic color sorting is a primary method for separating coffee beans suitable for production [15], this technique has yet to be explicitly tested for the separation of unripe beans in cultivation. Furthermore, recent studies show varying results when focusing on the separation of defective and non-defective green coffee beans based on chemical attributes. For instance, there are no significant differences in the proximal composition of green coffee [16] and unclear statistical differences in the fatty acid profiles of green coffee beans [17]. Additionally, there are no statistical differences in total levels of amines such as serotonin, spermidine, and spermine [18]. Some studies suggest that ESI-MS could be used to distinguish between immature and mature coffee beans [19]. However, the instrumental techniques used (such as chromatography or electrospray ionization mass spectrometry) and the associated analytical procedures are time-consuming and require significant manual effort [20].

Concerning remote sensing tools applied to coffee cultivation, research has shown that the spectral signature of coffee plants varies with age and throughout their phenological cycle [21]. Preliminary studies also indicate that the NIRS method is effective in determining the origin of green coffee from introgressed varieties [22]. We aimed to confirm whether it is possible to differentiate between varieties and/or degrees of maturity, contributing tools that enable improved selective harvesting, thus enhancing quality and market value, ultimately resulting in cost savings for producers [23]. Although designing a vision-based harvesting system for coffee cherries presents challenges (due to the size of the cherries, their cluster arrangement, and the height of the coffee plants [24]), recent advancements in satellite technologies and investments in a variety of sensors and satellite constellations by governments and private sectors are helping to overcome trade-offs in spectral, temporal, and spatial resolution.

The lack of accessible information in a common language during the coffee selection process for harvesting often leads to waste and financial losses. To address this issue, creating spectral libraries and quantifying colors for maturity grades is essential. This foundational work will facilitate the use of artificial intelligence to predict the degree of maturity, ultimately assisting farmers in making informed decisions through a software application. This research had two main objectives: (a) to generate spectral signatures for four coffee varieties (three with red fruits and one with yellow fruits) at eight stages of maturity, and (b) to develop predictive models for coffee maturity indices based on colorimetric data. The findings support the concept of using remote sensing combined with colorimetric pattern recognition to discriminate between the various degrees of maturity in coffee beans. This approach holds promise for enhancing post-harvest planning for specialty coffees by applying AI.

## 2. Materials and Methods

### 2.1. Study Area for Sample Collection

**Figure 1:**
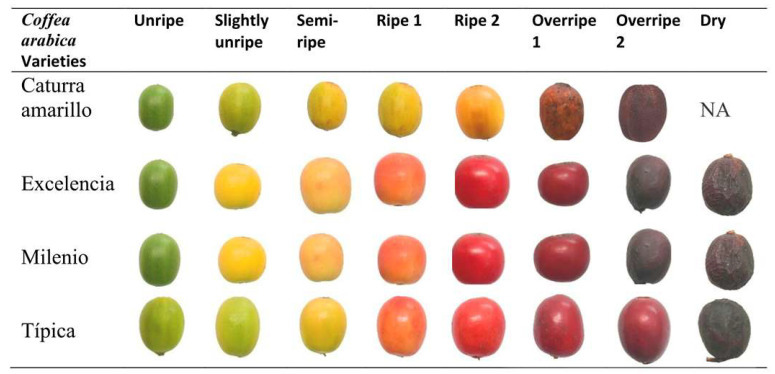
Varieties of *Coffea arabica* and degrees of maturity evaluated. NA: means that the variety does not exhibit maturity: Dry.

Samples of *Coffea arabica* were collected from plants at the Universidad Nacional Toribio Rodríguez de Mendoza de Amazonas, to facilitate variety registration. Four coffee varieties were grown in open fields in the Chachapoyas district, Chachapoyas province, Amazonas region, located at the geographic coordinates 6°13′59″ S 77°51′11″ W. The beans of each variety were visually classified into eight degrees of maturity for the red fruit varieties (Excelencia, Milenio, and Typica) as follows: Unripe, Slightly Unripe, Semi-Ripe, Ripe 2, Ripe 1, Overripe 2, Overripe 1, and Dry [25]. For the yellow fruit variety (Yellow Caturra), the beans were classified into seven degrees of maturity: Unripe, Slightly Unripe, Semi-Ripe, Ripe, Overripe 2, and Overripe 1 (Figure 1). A total of 20 grains of each color were used, and 10 repetitions of colorimetric analysis were performed on each grain.

### 2.2. Acquisition of Near-Infrared Spectral Images

For this study, we utilized a Parrot Sequoia multispectral camera (4608 × 3456 pixels, The sensor dimensions are 47 mm × 39.6 mm × 18.5 mm), which consists of four monochrome sensors that simultaneously capture four spectral bands: 550 nm ± 40 nm (green), 660 nm ± 40 nm (red), 790 nm ± 40 nm (near-infrared), and 735 nm ± 10 nm (RedEdge) [26,27].

The spectral images were captured under clear sky conditions between 11:00 AM and 1:00 PM local time. The multispectral camera was mounted on a tripod at a 90° angle to the plane of the samples. A fixed distance of 30 cm from the lens to the sample was maintained, a neutral background was used, and a guide was marked to center each fruit under the lens, following methodologies from similar studies [28]. The coordinates of the ground control points (GCP) were obtained using the GPS receiver integrated into the Parrot Sequoia multispectral camera [27,29]. Before and after each capture, images of the reflectance target provided with the multispectral camera were taken from a height of 1 m to perform radiometric calibration during post-processing [30]. Images were captured for all stages of grain maturity. The process of acquiring field images involved careful control of temperature and lighting to ensure consistent radiometric stability between captures. A calibrated halogen lamp with a color temperature of 5500 K was used to standardize lighting intensity, which helped reduce variability in reflectance caused by shadows or surface irregularities. Before each capture session, dark current calibration was performed to minimize sensor noise. Additionally, reflectance correction was applied using a Lambertian reference panel with known albedo coefficients. These procedures ensured that the spectral data remained reproducible across all maturity stages and varieties, thereby enhancing the reliability of the reflectance values used in later analyses. For each degree of maturity, one image was analyzed, and data quality control was implemented by excluding outlier pixels that deviated by more than ±2 standard deviations from the average reflectance within each spectral band.

### 2.3. Image Processing

All images were recorded and processed in Tagged Image File Format (TIFF). Image processing was carried out using ArcGIS Pro software version 3.4.2 (https://www.esri.com/en-us/arcgis/products/arcgis-pro/resources, accessed on 22 August 2025). Initially, the spatial reference of the images was corrected using WGS 1984 geographic coordinates [31]. Then, polygonal masks were manually created for each sampling point using QGIS software version 3.34 (https://qgis.org/, accessed on 22 August 2025). Subsequently, the average pixel values within the polygons were extracted using the zonal statistics tool [30].

### 2.4. Predictive Models Based on Colorimetric Data

Color Analysis

The color coordinates of green coffee beans, including L*, a*, and b*, were obtained using a colorimeter (CR-400, Konica Minolta, Inc., Tokio, Japan) at ten positions on the equatorial plane of the beans [32]. These measurements were based on the three-dimensional Cartesian space (xyz) of CIE L* a* b* color. L* represents lightness, ranging from 0 (black) to 100 (white) on the z-axis, indicating the lightness of the fruit color; the parameter a* represents redness (+) or greenness (−), while b* represents yellow (+) or blue (−) on the y-axis [33,34]. After verifying the suitability of the data set (KMO test = 0.75), a principal component analysis (PCA) was performed on the colorimetric parameters L*, a*, and b* for the Caturra Amarillo, Milenio, Excelencia, and Típica varieties to explore the variability related to the ripeness of the fruits.

Multiple Linear Regression Model (MLR)

Multiple linear regression methods are a modeling technique used to explain the impact of independent variables on a dependent variable [35], making them valuable for this research. This study employed the stepwise multiple linear regression technique [36], where *Y* represents the dependent variable, and β_1_, β_2_, β_3_, represent the independent variables, with L*, a*, b*, as the regression coefficients.

The MLR equation established in this study was derived from three variables, expressed in Equation (1).

(1) $$Y=constant+\beta_{1}\left(L^{*}\right)+\beta_{2}\left(a^{*}\right)+\beta_{3}\left(b^{*}\right)$$

In this equation, Y denotes the degree of maturity, β represents the coefficient calculated through regression, while L*, a*, and b*, represent luminosity, red–green coordinates, and yellow–blue coordinates, respectively.

The degrees of maturity of the four coffee varieties (yellow Caturra, Milenio, Excelencia, Típica) were used as predictor variables. To apply the MLR model, we examined the correlation between pairs of input variables to determine which to include. In the MLR model, it is standard practice to include input variables that have a high linear correlation with the response variable, while excluding those that are highly correlated with each other due to concerns about multicollinearity.

**Figure 2:**
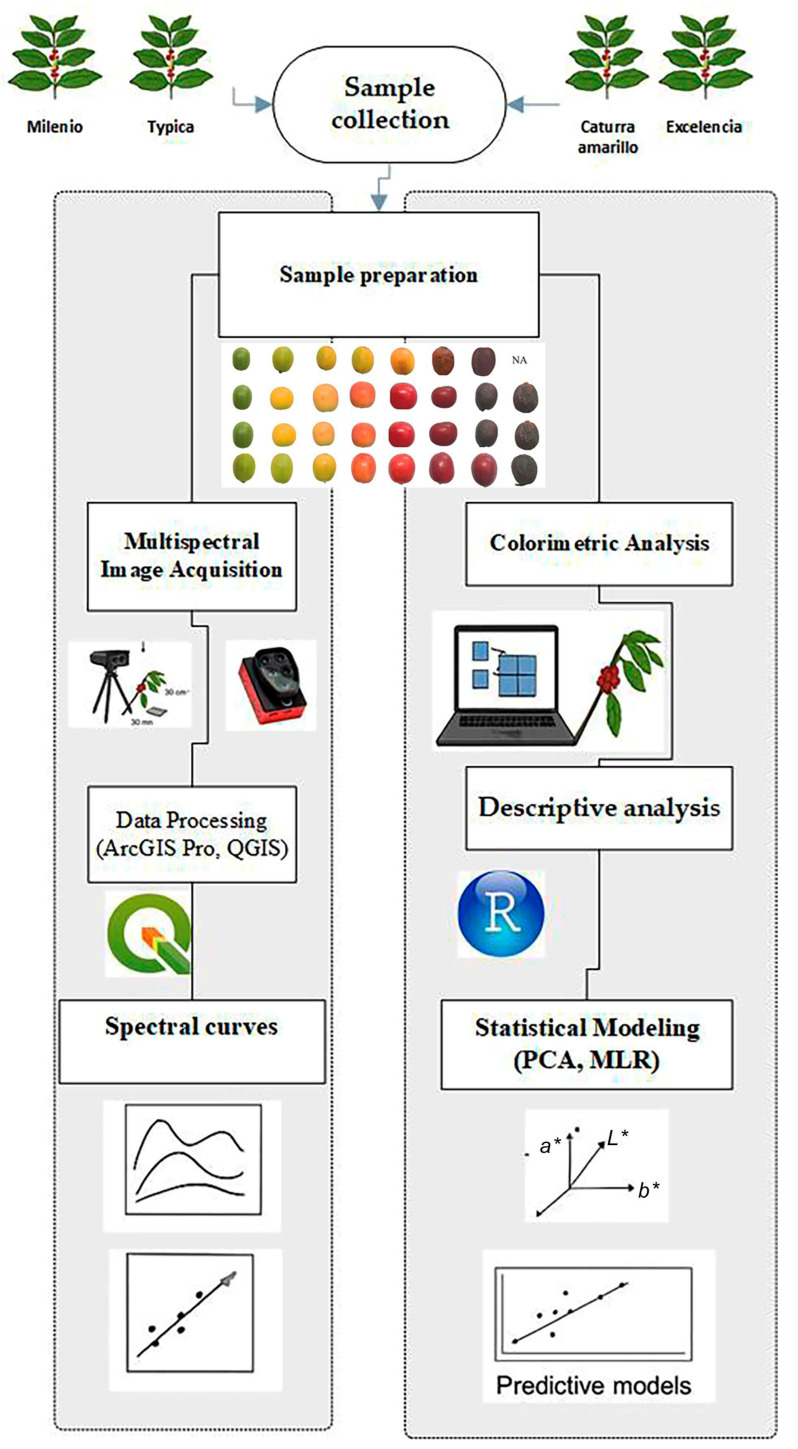
Schematic diagram of the experiment.

The Multiple R (multiple correlation coefficient) [37] was utilized to determine the correlation level between the observed values and those predicted by the model. Additionally, Multiple R^2^ (coefficient of determination, R^2^) [38] was used to measure the proportion of variance in the dependent variable that the independent variables in the model can explain. The *p*-value [39] was employed to assess whether the overall model is statistically significant. The model and statistical analysis were conducted using R version 4.5.1 (https://cran.r-project.org/bin/windows/base/, accessed on 22 August 2025), utilizing the following packages: stats, olsrr. A schematic diagram of the experiment is shown in Figure 2.

## 3. Results

### 3.1. Spectral Signature

Spectral analysis revealed consistent variations in the reflectance curves at different maturity stages for the four evaluated varieties of *Coffea arabica*: Yellow Caturra, Excelencia, Milenio, and Typica. In the green band (550 nm), reflectance was high in unripe fruits (0.52–0.60) and gradually decreased as the fruits became overripe (0.20–0.33). This decrease reflects the loss of green hues as the ripening process advanced. In contrast, the red band (660 nm) showed a gradual increase in reflectance from immature to mature stages (0.3–0.7), which is associated with the accumulation of carotenoid pigments and anthocyanins in the fruit’s epidermis.

**Figure 3:**
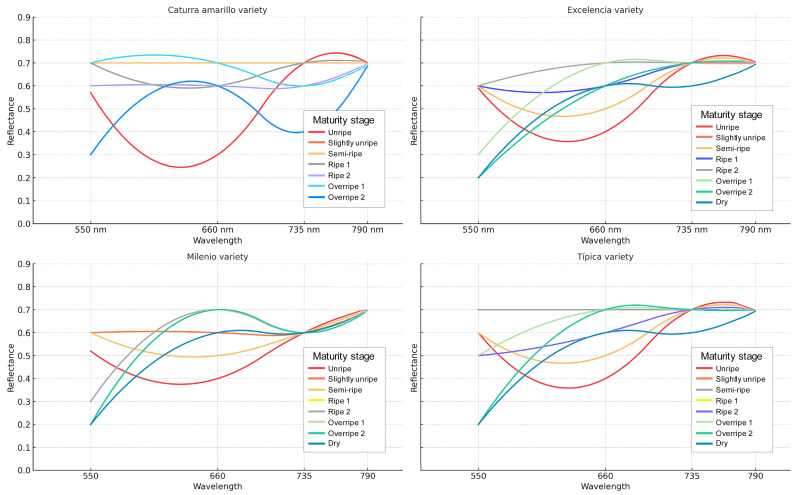
Spectral signature by maturity stage in Arabica coffee varieties.

Interestingly, the RedEdge band (735 nm) and the near-infrared region (790 nm) displayed consistently high reflectance across all ripeness stages (0.6–0.7). This indicates a less sensitive response to pigment changes but is linked to the cell structure and internal water content of the fruit. However, slight reductions in these reflectance values at overripe stages suggest physiological alterations in tissue integrity. These findings confirm that the visible bands (550 and 660 nm) are particularly informative for differentiating the phenological stages of coffee fruits. In contrast, the infrared bands (735 and 790 nm) offer complementary insights into the structural and physiological condition of the fruit. Together, these results provide robust support for using multispectral remote sensing in the non-destructive determination of coffee ripeness (see Table 1 and Figure 3).

### 3.2. Colorimetry Analysis

Analysis of colorimetry in the yellow Caturra variety revealed that green fruits exhibited high L* values (54.88 ± 3.56), negative a* values (−18 to −12), and low b* values (41.34 ± 4.07). As the fruit underwent intermediate ripening (from slightly unripe to semi-ripe and ripe), L* values increased slightly (66.95 ± 5.69), while a* values shifted towards slightly positive values (−11.99 to 3.35). Additionally, there was a marked increase in b* values (50.91 ± 6.35). In the overripe stage, lightness decreased (41.11 ± 7.81), a* values increased slightly (16.73 ± 4.03), and b* values reached a maximum (25.3 ± 11.05).

Similarly, in the initial stages (unripe and slightly unripe) of the Excelencia variety, the fruits demonstrated high brightness values (54.39 ± 1.86; 58.83 ± 2.84, respectively), negative a* values (−14.04 ± 3.05; −9.28 ± 5.13, respectively), and moderately high b* values (39.34 ± 3.32; 45.11 ± 3.16, respectively). As the fruits ripened (semi-ripe), there was a slight decrease in L* (57.95 ± 4.37), a notable increase in a* (18.29 ± 9.61), and a reduction in b* (43.06 ± 4.9). Full ripeness (Ripe 1 and Ripe 2) showcased a progressive decrease in brightness (45.51 ± 3.38; 40.36 ± 3.46, respectively). The high a* values (33.25 ± 4.2; 37.04 ± 2.01, respectively) indicated a spectral dominance of red and warm hues, whereas the b* component (26.57 ± 3.54; 19.71 ± 2.84, respectively) decreased significantly. In the overripe state, further reductions were observed in L* (32.45 ± 2.20) and b* (11.41 ± 2.99), while a* values remained high (31.04 ± 5.37), indicating warm reddish tones. Finally, in the dry stage, substantial decreases were noted in colorimetric values: L* (27.5 ± 1.58), a* (15.51 ± 5.1), and b* (5.16 ± 1.08).

For the Milenio variety, the initial unripe and slightly unripe stages reflected a lighter, greener color with L* (46.51 ± 1.12), a* (−14.38 ± 2.41), and b* (30.43 ± 1.5) components. In contrast, the semi-ripe and ripe stages indicated progressive darkening, as evidenced by the decrease in L* (54.43 ± 3.29; 53.96 ± 6.37, respectively), an increase in a* (−9.41 ± 6.74; 5.93 ± 7.58), indicating chlorophyll loss, and a marked increase in b* (40.76 ± 3.66; 39.21 ± 8.94), which reflects carotenoid accumulation. The overripe stage, characterized by darker and more saturated fruit tones, showed a moderate reduction in the L* (38.27 ± 2.7; 34.84 ± 2) and b* (18.63 ± 2.71; 14.5 ± 2.82) components. In the dry ripeness stage, considerable decreases were observed in L* (29.76 ± 1.81), a* (22.74 ± 6.06), and b* (9.61 ± 1.94), reflecting the effects of progressive dehydration during the drying process.

The Típica variety also comprises eight stages of ripeness. The initial unripe stage showed moderately high L* values (49.73 ± 2.52), negative a* values (−13.80 ± 5.28), and moderately high b* values (34.2 ± 1.83), indicating high chlorophyll content. As the fruits began to ripen (slightly unripe and semi-ripe), there was a notable increase in L* (58.14 ± 4.79; 63.22 ± 4.56), an increase in a* (−9.99 ± 4.97; 6.03 ± 7.94), and b* (44.71 ± 6.42). Full ripening (Ripe 1 and Ripe 2) was characterized by a progressive reduction in brightness (52.82 ± 5.52; 44.09 ± 3.93) and the b* component (33.87 ± 8.52; 23.62 ± 4.49), corresponding with the degradation of non-photosynthetic pigments. In the overripe state, further reductions in L* (42.31 ± 2.56; 37.41 ± 3.97) and b* (22 ± 2.68; 16.3 ± 4.18) were observed, while a* values remained high (37.89 ± 2.83; 36.97 ± 10.26), indicating warm reddish tones. Finally, in the dry state, there was a notable decrease in the colorimetric values: L* (30.31 ± 2.73), a* (25.37 ± 9.31), and b* (9.85 ± 2.96), resulting in a dark brown tone typical of the dehydration process (Figure 3).

### 3.3. Distribution of Ripeness States

**Figure 4:**
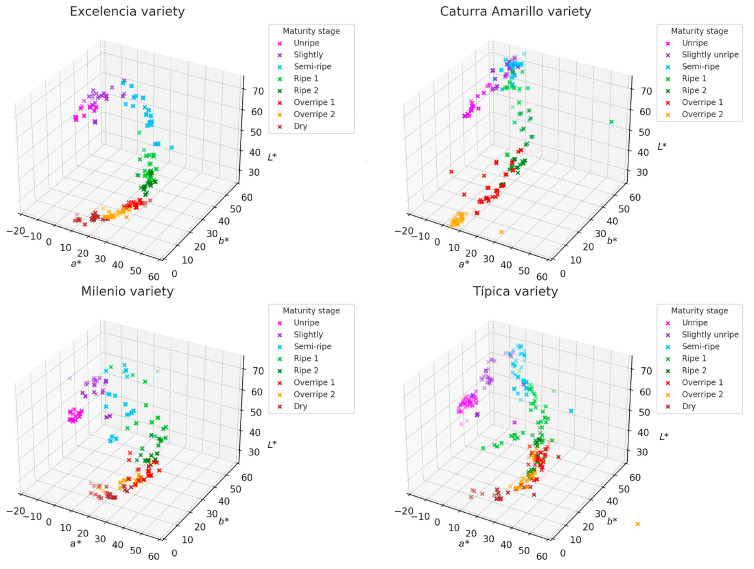
Distribution of the fruits of four coffee varieties based on the color parameters L*, a*, and b*.

Figure 4 illustrates the two-dimensional distributions (PC1 vs. PC2) along with the loading vectors corresponding to each parameter. The first two principal components accounted for more than 98% of the total variance across all varieties, confirming that the three colorimetric parameters are highly representative for describing the maturity of coffee fruits. This allows for the development of models for each variety. The vectors also indicate how the original variables (L*, a*, b*) impact the sample distribution: the a* vector has a strong projection on PC1, serving as the main discriminant in the transition from green to red. The L* vector is oriented in the opposite direction to PC1 in the Milenio and Típica varieties, suggesting that the darkening of the fruit (low L*) accompanies the ripening process. Though the b* vector (yellowness) has less influence, it remains relevant in varieties such as Caturra Amarillo and Excelencia, where ripe fruit takes on yellow tones.

**Figure 5:**
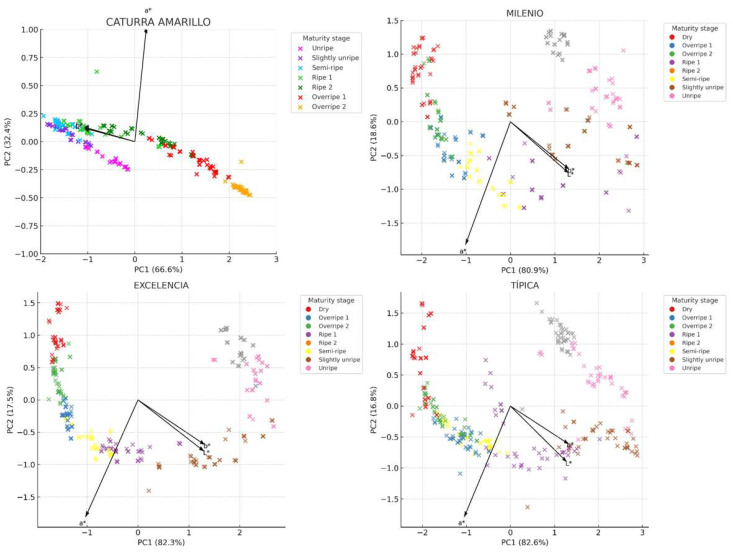
Principal component analysis (PCA) with loading vectors of colorimetric parameters (L*, a*, b*) in fruits of four Arabica coffee varieties.

Each stage of ripeness forms a distinct grouping in the principal component space, indicating a progressive evolution of color as the fruit develops. In all varieties, unripe fruits (categorized as Unripe and Slightly Unripe) cluster in regions with negative PC1 values, which are associated with higher brightness (L*) and greenish tones (negative a*). Ripe fruits (labeled as Ripe and Ripe 2) are located in the central or upper right part of the graph, corresponding to positive a* values (indicating a reddish tendency) and a decrease in L*. The stages of overripeness and drying (labeled as Overripe and Dry) show greater dispersion, likely due to chromatic heterogeneity resulting from pigment degradation (Figure 5).

### 3.4. Multivariate Linear Regression Modeling for Colorimetric Predictors

This section presents multiple linear regression models that predict the degree of coffee maturity using color variables in CIELab space (L*, a*, b*). All models demonstrate a *p*-value of 0.000, indicating that the color variables are highly significant predictors of maturity (*p* < 0.001). The R^2^ values range from 0.789 to 0.877, suggesting that these models can explain 78.9% to 87.7% of the variability in maturity. Notably, the Excelencia variety exhibits the highest R^2^ value (0.877), indicating a better fit compared to other varieties, whereas the Milenio variety shows the least accuracy with an R^2^ of 0.789.

Caturra Amarillo Variety (CAM)

The regression model for the Caturra amarillo variety (CAM) is as follows (Equation (2)):

CAM_Maturity = 3.664 + 0.039·L* + 0.101·a* − 0.064·b* (2)

In this formula, the constant and coefficients reflect the relative importance of each color component in predicting the degree of maturity for this variety. The multiple correlation coefficient (R^2^) indicates a high correlation (0.923) between the color values in the L*, a*, b* space at different ripeness stages and the predicted values from the model. An adjusted R^2^ value of 0.851 suggests that over 85% of the variability in maturity stages can be explained by the color variables considered. The model is statistically significant (*p* < 0.001). Among the coefficients, the a* parameter (green to red) shows the most important positive influence (0.101). This indicates that, although the Caturra amarillo does not exhibit visible reddish tones (since it is a yellow phenotype), even yellow phenotypes undergo physiological development visible in color changes, during ripening. The L* component (lightness) has a moderate positive effect (0.039), suggesting that the gradual lightening of the fruit is also an indicator of its physiological development. In contrast, the b* component (blue to yellow) has a negative coefficient (−0.064), implying that although the fruit acquires a more yellow hue at visual maturity, the change in b* is not linear and may be influenced by variations in surface reflectance and carotenoid accumulation. These results support the use of colorimetric models as non-destructive tools for objectively classifying coffee fruit maturity.

Milenio Variety (MI)

The regression model for the Milenio variety is (Equation (3)):

MI_Maturity = 10.048 + (−0.17·L*) + 0.059·a* + 0.037·b* (3)

This model demonstrates acceptable predictive power for estimating the degree of fruit ripeness based on color parameters. With an adjusted R^2^ of 0.787 and a multiple correlation value (R = 0.888), there is a statistically significant relationship between the observed and estimated values (*p* < 0.001). The equation indicates that changes in lightness (L*), redness (a*), and yellowness (b*) explain approximately 78.7% of the variability observed in the ripeness of Milenio fruits. The negative coefficient for the L* component (−0.17) suggests that ripening in the Milenio variety results in a considerable loss of lightness, which is related to chlorophyll decomposition and the progressive darkening of the fruit. Conversely, the positive coefficient for a* (0.059) indicates a gradual transition towards reddish tones, consistent with pigment synthesis (e.g., anthocyanins) during ripening. The b* parameter, with a coefficient of 0.037, suggests that the increase in yellowness also contributes, albeit to a lesser extent, to predicting ripeness. The combination of these factors reinforces the utility of colorimetric models as objective tools for non-destructive ripeness discrimination, particularly for varieties such as Milenio, where color change is progressive and multifactorial.

Excelencia Variety (E)

The regression model for the Excelencia variety is (Equation (4))

E_Maturity = 8.753 + (−0.05·L*) + 0.014·a* + (−0.093·b*) (4)

This model exhibits robust performance in predicting the degree of maturity with an adjusted R^2^ of 0.876 and a high multiple correlation value (R = 0.936). These values indicate that the model can explain 87.6% of the variability in fruit ripeness based on the colorimetric coordinates in CIELab space. The high statistical significance (*p* < 0.001) supports the model’s validity for practical non-destructive classification. The b* component (yellowness) has the most excellent absolute coefficient (−0.093), suggesting that the ripeness of Excelencia fruits is inversely related to this parameter. As the fruits ripen, the intensity of the yellow tone decreases, likely due to carotenoid degradation and the emergence of secondary pigments. The L* component also has a slight negative relationship (−0.05), indicating a progressive decrease in lightness associated with maturity. Overall, these models highlight the potential for colorimetric approaches in evaluating coffee fruit maturity in a non-destructive manner across different varieties.

Típica variety (T)

The Típica variety (T) is described by the following equation for maturity (Equation (5)):

T_Maturity = 8.848 + (−0.14·L*) + 0.067·a* + (−0.039·b*) (5)

The multiple linear regression model developed for the Típica variety demonstrates strong predictive capability, with an adjusted R^2^ of 0.816. This indicates that 81.6% of the variability in maturity grades can be explained by the colorimetric values L*, a*, and b*. Additionally, the Multiple R value of 0.904 shows a high correlation between the predictions from the model and the values observed through experimentation. The statistical significance (*p* < 0.001) further confirms the model’s validity for practical applications. This formula enables the accurate estimation of maturity levels in this variety using non-destructive techniques.

Analyzing the coefficients from the equation reveals interesting insights. The L* parameter (lightness) has a significant negative influence of −0.14, suggesting that maturity in Típica is linked to a progressive darkening of the fruit. This darkening is likely due to the degradation of chlorophylls and the synthesis of darker pigments, such as anthocyanins. The a* component, with a positive value of 0.067, indicates a shift towards reddish tones during ripening, which aligns with the visual characteristics of this variety as it develops vibrant colors in its later stages. In contrast, the b* coefficient, which has the smallest magnitude at −0.039, suggests that yellowness plays a minor role in the ripening process.

Overall, these findings illustrate that the Típica variety undergoes a chromatic transformation characterized by a loss of brightness and an intensification of red hues. These factors are essential for the development of automatic classification optical systems.

## 4. Discussion

Generation of Spectral Signatures

The spectral characterization of *Coffea arabica* fruits revealed consistent patterns between different stages of maturity, confirming that reflectance in the visible region (specifically green and red) is closely linked to the dynamics of photosynthetic pigments that capture light energy between 400 and 650 nm [40]. In the immature stages, high reflectance values at 550 nm indicate a strong presence of chlorophylls. As the fruit matures, these reflectance values gradually decrease, reflecting the degradation of these pigments [41]. Concurrently, an increase in reflectance at 660 nm is associated with the emergence of carotenoids and anthocyanins [30], making this band a sensitive marker for the fruit’s color transition [38]. These findings support the notion that the visible region of the spectrum is essential for accurately discriminating the stages of maturity in coffee.

In contrast, the relative stability of the RedEdge (735 nm) and near-infrared (790 nm) bands throughout the ripening process suggests that these wavelengths are less sensitive to changes in surface pigments and are more indicative of internal structural and physiological properties [41]. The red region of the fluorescence emission is subject to reabsorption, as it overlaps with the chlorophyll absorption spectrum, while the far-red portion is less affected [42]. The slight reduction in reflectance observed in overripe fruits may be linked to cellular senescence processes, loss of turgidity, and modifications in the cell wall. These results align with recent studies that highlight the potential of the RedEdge and NIR bands to monitor physiological conditions and water stress in crops [43], extending their application beyond merely classifying visual maturity in coffee.

The combination of these spectral responses presents opportunities to develop non-destructive, low-cost methodologies that integrate multispectral sensors into selective harvesting systems or aerial platforms [44]. The clear differentiation between immature, mature, and overripe states supports the potential to train machine learning algorithms with reference spectral signatures, enhancing real-time classification processes. This approach could reduce reliance on subjective manual selection methods, thereby improving the efficiency of the coffee value chain and ensuring greater consistency in cup quality.

Ultimately, the differences between varieties in the magnitude and direction of spectral changes highlight the necessity to calibrate specific models for each cultivar in future research [14,45]. For instance, Yellow Caturra exhibited a greater dependence on the yellow component (b*). At the same time, Milenio and Typica displayed more pronounced changes in the a* vector, associated with the green–red transition. This genetic and phenotypic variability indicates that, while spectral signatures are highly informative, their integration into predictive models must consider the specific characteristics of each variety and agroecological context [46]. Therefore, developing spectral libraries tailored to each production area is a crucial strategy to enhance the accuracy and transferability of these tools.

Field studies on spectral signatures of coffee demonstrate that variability in spectral signatures at the plant level begins in the 685 nm region of the spectrum [47], while expression at the maturity stage shows changes across all spectra in every maturity stage [30]. Although previous research has been demonstrated that PCA (Principal Component Analysis) in NIR spectra effectively distinguishes between coffees from different collections [22], advancements based on these research results support the use of PCA to identify colorimetric parameters (L*, a*, b*) in the fruits of four Arabica coffee varieties.

Colorimetry and Models

The colorimetric results (L*, a*, b*) indicate that the a* parameter is the primary indicator of maturity across all varieties, as it reflects the green-to-red transition associated with the degradation of chlorophylls and the synthesis of carotenoids and anthocyanins. This finding is consistent with earlier studies on coffee [30,34]. The L* value decreased as the fruit ripened, confirming the gradual darkening linked to senescence and the accumulation of dark pigments [33]. Additionally, the b* parameter played a varied role: it was crucial for assessing maturity in Caturra Amarillo and Excelencia, while in Típica and Milenio, its relevance was lower. This variation highlights the genetic differences in color dynamics among the varieties [46]. Principal Component Analysis (PCA) revealed a clear distinction between immature, mature, and overripe states in the multivariate space, suggesting the potential for integrating computer vision and machine learning algorithms into selective harvesting practices [9,11].

Multiple Linear Regression (MLR) models and adjusted R^2^ values demonstrate the strong predictive capability of colorimetric parameters. The best fit was found in Excelencia (R^2^ = 0.877), where the change in b* was critical, while Milenio exhibited lower accuracy (R^2^ = 0.789), likely due to greater chromatic heterogeneity. Coefficient analysis indicated that while each variety displayed distinct dynamics (for instance, loss of brightness in Typica and changes in b* in Excelencia), all models were statistically significant (*p* < 0.001). These findings have direct implications for the specialty coffee industry, as they minimize subjectivity in fruit selection and enhance batch homogeneity, thus improving quality and market value. However, intra-fruit variability and the influence of water status can impact accuracy. Therefore, future studies should incorporate measurements of moisture and physiological stress alongside color parameters [19,43].

In summary, colorimetry in the CIELab space and MLR models are effective, non-destructive, and cost-efficient tools for predicting ripeness. However, they require varietal calibration and validation across different agroecological contexts.

Limitations and Future Perspectives

The combination of multispectral imaging and colorimetry has proven effective for non-destructive maturity assessment of *Coffea arabica*. However, environmental factors such as light intensity, humidity, and background interference can significantly affect spectral responses and colorimetric readings. Additionally, the analysis was limited to four Arabica varieties grown in a single agroecological zone of the Peruvian Amazon, which restricts the ability to generalize the results to other regions or cultivars with different pigmentation dynamics and canopy structures. Another limitation stems from the static acquisition of multispectral images using a ground-based setup. While this configuration allowed for precise spectral calibration, it does not account for the complexity of aerial or drone-based imaging systems typically used in large-scale monitoring. Therefore, future research should validate these findings under dynamic conditions by incorporating UAV-mounted multispectral sensors, machine vision algorithms, and real-time image correction models. Moreover, integrating near- and mid-infrared spectroscopy could enhance the sensitivity to internal physiological parameters, such as moisture content and chlorophyll degradation. Looking ahead, there are opportunities to expand the spectral library by collecting samples from multiple growing regions, diverse coffee genotypes, and various environmental gradients. Integrating the developed models with artificial intelligence and cloud-based data management systems could create predictive tools for automated coffee cherry sorting and selective harvesting. Ultimately, the combination of multispectral imaging, colorimetry, and AI-driven analytics presents a promising pathway for precision agriculture, aimed at improving quality control, optimizing harvest timing, and enhancing sustainability within the specialty coffee value chain.

## 5. Conclusions

The results indicate that colorimetry in the CIELab color space (L*, a*, b*) is an effective tool for distinguishing between different stages of ripeness in *Coffea arabica* fruits. Principal Component Analysis (PCA) revealed consistent patterns of color transitions across all varieties, showing a clear separation among immature, mature, and overripe fruits. Notably, the a* parameter primarily differentiates the green–red transition, while L* and b* provide supplementary information related to darkening and the accumulation or degradation of pigments.

This non-invasive classification of maturity stages opens up the possibility of integrating artificial vision and remote sensing technologies into selective harvesting practices. This integration can minimize subjectivity and enhance the quality of the harvested coffee.

Furthermore, Multiple Linear Regression (MLR) models exhibited strong predictive capabilities, with adjusted R^2^ values ranging from 0.789 to 0.877. This indicates that over 78% of the variability in coffee maturity can be explained by color parameters. The Excelencia variety yielded the best model performance, with the b* component playing a crucial role in estimating ripeness. In contrast, the Milenio variety displayed greater chromatic variability.

These findings suggest that while colorimetric models are highly effective, they necessitate specific calibrations for each variety and should be validated across different agroecological conditions. Overall, the combination of colorimetry and statistical modeling presents a non-destructive, accessible, and transferable approach to optimizing the classification of coffee cherries, with significant potential applications in the specialty coffee industry and intelligent harvesting support systems.

## Figures and Tables

**Table 1:** Spectral reflectance curves for different ripeness stages in fruits of four coffee varieties (Yellow Caturra, Excelencia, Milenio, and Typica), measured in the multispectral bands: 550 nm (Green), 660 nm (Red), 735 nm (RedEdge), and 790 nm (NIR).

Variety/Wavelength (nm)	Unripe	Slightly Unripe	Semi-Ripe	Ripe 1	Ripe 2	Overripe 1	Overripe 2	Dry	Band
Caturra amarillo/550	0.57	0.7	0.7	0.7	0.6	0.7	0.3	-	Green
Caturra amarillo/660	0.3	0.6	0.7	0.6	0.6	0.7	0.6	-	Red
Caturra amarillo/735	0.7	0.7	0.7	0.7	0.6	0.6	0.4	-	RedEdge
Caturra amarillo/790	0.7	0.7	0.7	0.7	0.7	0.7	0.7	-	NIR
Excelencia/550	0.59	0.6	0.6	0.6	0.6	0.3	0.2	0.2	Green
Excelencia/660	0.4	0.5	0.5	0.6	0.7	0.7	0.6	0.6	Red
Excelencia/735	0.7	0.7	0.7	0.7	0.7	0.7	0.7	0.6	RedEdge
Excelencia/790	0.7	0.7	0.7	0.7	0.7	0.7	0.7	0.7	NIR
Milenio/550	0.52	0.6	0.6	0.3	0.3	0.2	0.2	0.2	Green
Milenio/660	0.4	0.6	0.5	0.7	0.7	0.7	0.7	0.6	Red
Milenio/735	0.6	0.6	0.6	0.6	0.6	0.6	0.6	0.6	RedEdge
Milenio/790	0.7	0.7	0.7	0.7	0.7	0.7	0.7	0.7	NIR
Típica/550	0.6	0.6	0.6	0.7	0.5	0.5	0.2	0.2	Green
Típica/660	0.4	0.5	0.5	0.7	0.6	0.7	0.7	0.6	Red
Típica/735	0.7	0.7	0.7	0.7	0.7	0.7	0.7	0.6	RedEdge
Típica/790	0.7	0.7	0.7	0.7	0.7	0.7	0.7	0.7	NIR

## Data Availability

The original contributions presented in the study are included in the article, further inquiries can be directed to the corresponding author.
